# Tree-ring width reveals the preparation of the 1974 Mt. Etna eruption

**DOI:** 10.1038/srep44019

**Published:** 2017-03-07

**Authors:** Ruedi Seiler, Nicolas Houlié, Paolo Cherubini

**Affiliations:** 1 Swiss Federal Institute for Forest, Snow and Landscape Research WSL, Zürcherstrasse 111, 8903 Birmensdorf, Switzerland; 2 Department of Geography, University of Zurich, Winterthurerstrasse 190, CH-8057 Zürich, Switzerland; 3 SEG - IFG, Eidgenössische Technische Hochschule (ETH), 8093 Zürich, Switzerland; 4 MPG – IGP, Eidgenössische Technische Hochschule (ETH), 8093 Zürich, Switzerland

## Abstract

Reduced near-infrared reflectance observed in September 1973 in Skylab images of the western flank of Mt. Etna has been interpreted as an eruption precursor of the January 1974 eruption. Until now, it has been unclear when this signal started, whether it was sustained and which process(es) could have caused it. By analyzing tree-ring width time-series, we show that the reduced near-infrared precursory signal cannot be linked to a reduction in annual tree growth in the area. However, comparing the tree-ring width time-series with both remote sensing observations and volcano-seismic activity enables us to discuss the starting date of the pre-eruptive period of the 1974 eruption.

Early detection of precursors to volcanic eruptions is important in reducing risks for populations and damage to infrastructures^1^,^2^,^3^,^4^,^5^,^6^,^7^,^8^. Once a volcanic eruption has started, thanks to modern techniques, we are now able to describe both seismic activity^1^,^2^,^3^,^4^,^5^,^6^,^7^,^8^ and surface deformation^9^,^10^,^11^,^12^ to constrain the origin of magma through gas changes^13^,^14^,^15^ and to understand how magma intrusions propagate^16^,^17^. Still, we know too little about how the magma moves under the surface during pre-eruptive periods (months to days before the magma reaches the surface). For instance, it is sometimes unclear if magma input from depths is steady^18^, or, for how long magma could be stored at shallow depths (and eventually degassed) without being detected by seismic and geodetic networks. If not recorded because of their unconventional or too tenuous appearance, precursors of volcanic activity are lost forever.

Following the studies dedicated to vegetation monitoring from space, we propose to use trees to monitor volcanic activity. Trees are likely not reacting directly to volcanic activity but as it has been proposed they may respond to associated environmental changes^19^,^20^,^21^,^22^ such as water table variation, gas emissions, atmospheric water vapor or sudden temperature changes. As tree growth largely depends on environmental conditions (i.e., on the local availability of water and nutrients, and temperature during the vegetation season) tree rings have been widely used in the environmental sciences as proxies to assess both local and regional past climate variations^23^,^24^,^25^ or photosynthesis rates^26^. As trees form one growth ring each year, tree-ring chronologies may be used as annually resolved, long-term records of past eruptive events. Recently, it has been shown that pre-eruptive volcanic activity occurring during the vegetation period can influence the photosynthesis of trees, as shown by Normalized Difference in Vegetation Index (NDVI) derived from satellite imagery months before the beginning of the 2002–2003 eruption of Mt. Etna and the 2002 eruption of Mt. Niyragongo^20^. These observations were not the first satellite observables of this kind. Before the time of modern remote sensing sensors, a reduced near-infrared (NIR) signal was detected in September 1973 in the area of Monte de Fiore (Fig. 1) prior to the 1974 Mt. Etna eruption^27^. In this case, the start and duration of the NIR signal could not be determined nor was the cause of the signal identified as the eruptive activity started months later. In order to test whether trees were impacted by volcanic activity and with the aim of better understanding the dynamics of the early stages of the 1974 eruptive event, we built ring-width time-series of trees located near Monte de Fiore.

The 1974 eruption is key in the recent eruptive history of Mt. Etna when investigating how the magma feeding systems works. The eruption of January–March 1974 represents a petrological transition between two eruptive periods during which different types of magma were emitted: a phenocryst-charged basalt type, which was observed coming from the central vent until 1974, and a more glassy basalt type, free of plagioclase that has been observed in many occasions since 1974^28^,^29^ and at least until the 2001–2003 flank eruption^30^. According to Rittman’s classification^31^, the 1974 eruption was either eccentric^32^, eccentric with deep origin^30^,^33^ or lateral^30^,^34^. Because of the lack of phenocrystals in the emitted basalts^33^, it has been proposed that the magma quickly ascended^33^ from a source located 11 km below ground^30^, corresponding to the depth at which the Mt. Etna magma chamber has been constrained^12^,^35^,^36^.

The 1974 eruption (30 January 1974–March 1974) was preceded (~10 days) and accompanied by one of the greatest seismic crisis ever observed on Mt. Etna^34^,^37^ with up to 150 events per day detected at the Mt. Vetore station (20 events per day at the Catania station; Fig. 2 and Material and Methods section). For reference, during the 1971 crisis, the daily seismic rate did not exceed 10 events per day at the Catania station^38^. The occurrence of seismic crisis before an eruptive period is not surprising. It is well known that seismic activity precedes volcanic activity^39^,^40^,^41^,^42^,^43^, also on Mt. Etna^3^,^37^,^40^,^44^,^45^,^46^,^47^ and afterwards is often associated with magma intrusions. Before the onset of the January 1974 eruption, Mt. Etna was already seismically active. In the summer-autumn 1973, a seismic event triggered the deployment of a seismic network on Mt. Etna^48^. Unfortunately, due to technical issues, the locations of the October events, could not be determined with certainty. The temporal coincidence of the October 1973 seismic crisis with both renewed activity at the Voragine and the NIR signal, raises the question of the presence of an early intrusion into the western flank in the vicinity of Monte de Fiore.

In this study, we (i) evaluate whether the ring-width time series of Monte de Fiore trees changed before and after the eruption, (ii) assess whether the NIR signal detected^27^ can be associated with tree-ring growth changes, and (iii) discuss our findings in the context of geophysical observations made between September 1973 and March 1974.

## Results

**Figure 1:**
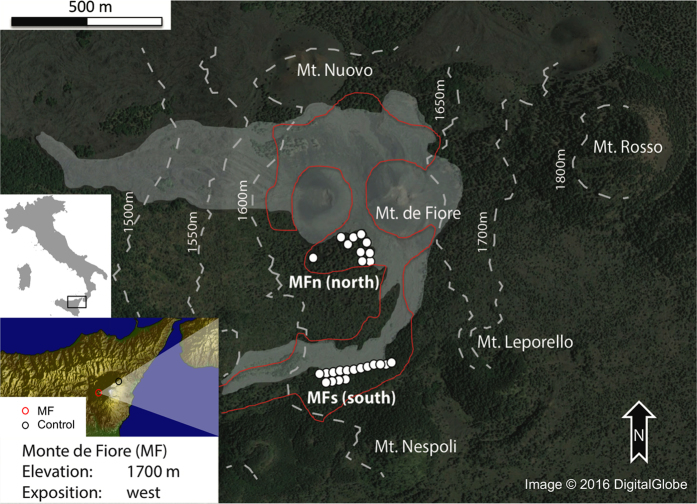
Monte de Fiore sample locations. Local aerial view of Monte de Fiore on the western flank of Mt. Etna near the 1974 vent. Location of trees sampled are marked with white dots, the area of the NIR signal^27^ with a red line, and the 1974 lava flow with grey shading. The map of Italy was created using the program R (Version 3.1.3; URL: http://www.R-project.org/)^60^ and the topographic map showing Sicily was created using Generic Mapping Tools (Version 5.2.1; URL: http://gmt.soest.hawaii.edu/)^61^. The main map showing the Monte de Fiore area was derived from Google earth (Google, DigitalGlobe, 2016)^62^ supplemented with contour lines^63^ displaying topography.

We sampled two groups of trees: MFs, south of the southernmost 1974 lava flow, and MFn, in the north close to the Monte de Fiore craters (1737 m a.s.l.). All trees were growing in the area where the NIR signal disturbance was observed^27^ (see the red contour line in Fig. 1). In total, we collected 52 cores from 26 trees (*Pinus nigra* J. F. Arnold) growing in the area close to the 1974 eruption at Monte de Fiore (MF). MFn trees are located very close to the 1974 eruptive craters and include 10 trees, whereas the 16 trees of MFs are farther away along the lava flow that originated from Monte de Fiore (Fig. 1). Since the trees were located close (<1 km) to the Monte de Fiore craters, their growth was probably disturbed by projectiles, lava-flow heat^49^ and fires during the 1974 eruption. We expected MFn trees to be more disturbed than the MFs trees as they were closer to the 1974 vents. In order to establish the growth of an undisturbed control group, we sampled the cores of 50 control trees of the same species, located on the north-eastern flank of Mt. Etna 13 km away close to Piano Provenzana (control group) at an elevation of approximately 1700 m a.s.l. The growth of these trees was probably not disturbed by volcanic activity during the 1970s and 1980s because there was little eruptive activity during this period along the North-East rift.

### Tree-ring growth

Most of the trees in MFn germinated in the 1950s and are younger than trees in MFs, which mostly germinated between 1910–1920 (Supplementary Fig. S1). The similar tree ages in each of the two groups suggest that the germination of the trees probably happened after disturbances such as wildfire or clear cutting.

**Figure 3:**
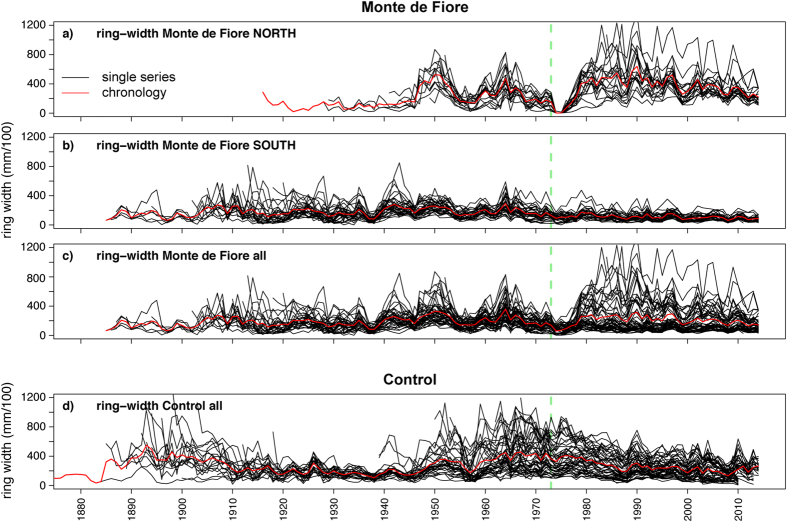
Raw tree-ring chronologies. Ring-width chronologies for trees in MFn (**a**), MFs (**b**), both parts of Monte de Fiore (**c**) and the control site (**d**). The vertical green line indicates the time of the eruption. For most MFn trees, which were closest to the vent, growth stopped for two consecutive years following the 1974 eruption. Such an impact is not visible in the tree-ring width time-series of MFs trees. A zoom image for the eruptive period of this figure is available in the Supplementary Materials (Supplementary Fig. S4).

Nine out of ten trees in MFn did not form any rings during two consecutive vegetation seasons (1974 and 1975; Fig. 3a), probably because of the heat radiating from the lava. Trees at other sites on Mt. Etna have also been found to lack tree rings close to lava flows^49^, and we explain these observations as only one hour of exposure to temperatures of 60 °C is able to decrease tree growth^50^. In contrast, trees in MFs located close to a lava flow but far away from the crater formed regular rings during 1974 and 1975 and seemingly were not affected by the heat emitted by the lava flow (Fig. 3b). Before comparing the tree-ring width of 1973 with the ring width of previous years, we need to correct for the age-related growth trend in trees. Only then will it be possible to discuss whether the observed reduction of photosynthesis was already sustained before September 1973.

### Age-growth relationship

**Figure 4:**
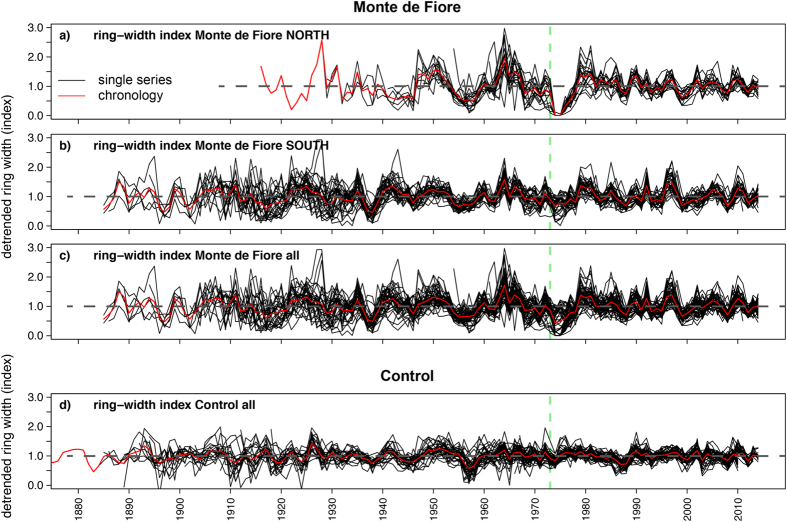
Detrended tree-ring chronologies. Same data as in Fig. 3 after applying detrending to samples from MFn (**a**), MFs (**b**), all of Monte de Fiore (**c**) and the control site (**d**). After correction for the age trend, the growth increase of the MFn trees after 1976 is no longer visible. The vertical green line indicates the time of the eruption.

Tree growth varies over the lifespan of a tree. Such ontogenetic variation strongly impacts the shape of the raw tree-ring width time-series, an effect also known as “age trend”^51^. For dendrochronological purposes, age trends are removed using standardizing methods depending on what growth information is needed^52^. In this study, we used a 30-year spline detrending where a “moving window” is used to standardize the raw data series^51^ together with a variance stabilization to obtain equally balanced chronologies which preserved the short-term variability^52^,^53^. In Fig. 4 the detrended ring-width data for trees in MFn (a), MFs (b), both parts of Monte de Fiore (c) and the control site (d) are shown. After detrending the low-frequency variability of the raw time series is eliminated but high-frequency variability, such as the reduced growth of MFn trees during 1974–1975, is preserved.

**Figure 5:**
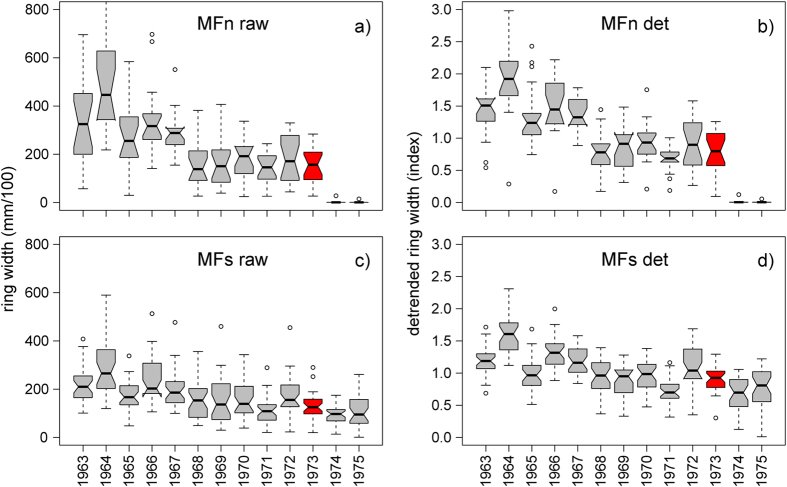
Ring-width comparison. Boxplots showing the median, lower and upper quartiles and the minimum and maximum tree-ring widths of Monte de Fiore samples (raw data [**a**,**c**] and detrended data [**b**,**d**]) in both MFn and MFs. The ring width in MFn and MFs of 1973 is highlighted in red and does not statistically differ (t-test; p > 0.05) from the ring width between 1968 to 1972. This indicates that tree growth there was not reduced before the eruption. The ring width of MFn samples was strongly influenced in 1974 and 1975. The reductions in width of both MFn and MFs samples were statistically significant in 1974 and at MFn in 1975 too (t-test; p < 0.001).

Tree-ring patterns are affected by changes in the forest stand structure that changes competition processes for resources such as water and light^54^,^55^. The tree-ring width patterns of the sampled trees might be affected by changes in the stand during the past decades, e.g., those induced by the death of trees. However, the ring-width patterns of our samples do not show any evidence of changes in competition, e.g., abrupt growth changes except for the growth release of MFn trees during the time after the 1974 eruption, most likely effects of reduced competition caused by burning of some trees After correction for the age trend, the erratic growth of MFn trees and the slow decrease in the growth of MFs trees after 1974 in the raw ring-width data are no longer visible (Fig. 4). Comparing the ring widths of 1973 and 1974–1975 with those of the preceding years 1968–1972 (Fig. 5) suggests that none of the trees showed disturbed growth during 1973, but the growth was strongly reduced or completely suppressed in MFn in 1974 and 1975. Thus, tree growth in this area was significantly impacted only after the eruption (t-test; *p* < 0.01).

### Response to climate

The internal correlation coefficient (inter-correlation) for the Monte de Fiore series is 0.6 (Table 1), which suggests that all trees were growing synchronously. Furthermore, the patterns in tree growth in Monte de Fiore (MF) and Piano Provenzana (control; north-eastern rift zone; inter-correlation = 0.53) were similar. Over the last 100 years from 1915 to 2014, the correlation between MF and control age-detrended ring width was 0.5 (*p* < *0.001*, Supplementary Fig. S2) despite the sites having different expositions and being approximately 13 km apart. This inter-group correlation suggests that tree growth in MF and at the control site during this period (Supplementary Fig. S2) was mostly influenced by common factors (i.e. weather, climate, nutrient availability, volcanic activity).

To quantify the impact of climate variations on tree growth, we used interpolated temperature and precipitation anomalies (1924–2004) from the CRU databank (Climatic Research Unit, University of East Anglia, Norwich, U.K.)^56^,^57^ and compared them to tree-growth patterns. Tree-growth at similar elevations on Mt. Etna was shown to be weakly influenced by climate variability^58^. We found that only variations in July precipitation significantly affected trees in MF (r < 0.23; *p* < *0.05*). This effect, however, was weak. Climate had a stronger influence on the control chronology (Supplementary Fig. S3), with positive precipitation-ring width correlations during summer (0.22 < r < 0.35; *p* < *0.05*), and temperature-ring width correlations in August (r = −0.4; *p* < *0.001*) and March (r < 0.35; p < 0.01). The rather low correlations are supported by observations in Mediterranean area at similar elevations^59^. Temperature and precipitation thus do not seem to strongly affect tree growth at Monte de Fiore. No eruption precursory signals could be found in the tree-growth time-series (Fig. 5c and d). This suggests that the NIR signal observed from SKYLAB was either i) not sustained before September 1973 and therefore had no impact on tree growth, or ii) was sustained but occurred after the vegetation period.

### Response to volcanic activity

Consequences of pre-eruptive volcanic activity on tree growth strongly vary with the nature of the processes in place. Volcanic activity has been associated with enhanced photosynthesis during pre-eruptive periods^27^. In other contexts, as in the Mammoth mountains in 1990s^60^ and possibly also in Pico del Nambroque, La Palma in 1949^61^, pre-eruptive volcanic activity led to tree damage or even die-off. Similarly, the observed decrease in NIR reflectance on the western flank of Mt. Etna^27^ may have been caused by processes associated with the inception of the 1974 event. In September 1973 at the time of the SKYLAB observation^62^, the NDVI processing technique had not yet been developed^63^. Because only the NIR band was available, it is challenging to identify the nature of the process responsible for the detected signal. If the NDVI method had been used with both red and infrared bands, it would have been possible to distinguish between changes of vegetation canopy reflectance and ground temperature.

Growth analyses on trees growing at latitudes comparable to Mt. Etna have shown that tree growth stops after August even though photosynthesis may still be active^64^. Then, if precursory activity had changed the ecological settings around the trees during the growth period of 1973 (March–August 1973), associated signals would be visible in the ring-width time-series. The reduced NIR signal observed with SKYLAB in 1973 may have been caused by higher soil moisture^65^,^66^ due to degassing of large amounts of volatiles^67^,^68^ or a decrease in the photosynthetic activity in the area^27^,^69^,^70^. The 1973 ring widths do not differ statistically (t-test; *p* > *0.1*) from those of the previous five years (1968–1972) as discussed earlier. If a change in volcanic activity affected tree growth in the area, it must have started at the end of, or after, August 1973. However, the end of the detected NIR signal cannot be constrained in time using tree-ring width analysis as trees are not reacting to environmental influences after the end of the growth season. Here seismic activity might help, as the seismic event of 1973 lasted until the end of October. This date might then be the end of the magma intrusion. At last, in the light of observations collected/comprised in this study, we suggest that the SKYLAB NIR anomalous signal may have been the result of an early dyke intrusion, initializing the 1974 eruptive event. Observed seismic^34^,^37^ and explosive (at central crater) activity during the months of September and October 1973^34^ (see Material and Methods section) further support this hypothesis and may help dating the end of the intrusive episode.

## Conclusion

**Figure 6:**
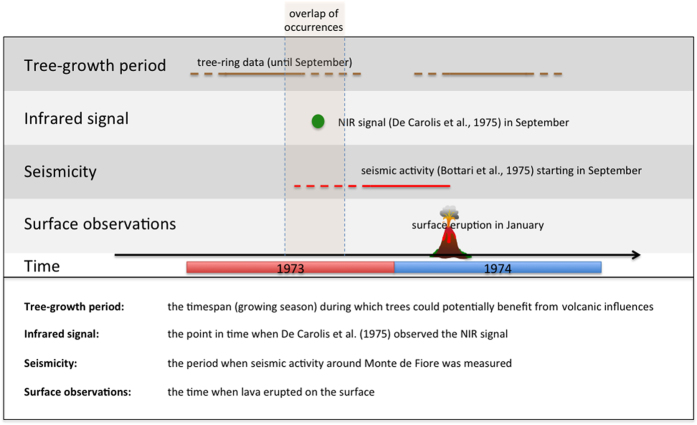
Activity observations in time. Timeline of observations available (tree growth, NIR, seismicity and surface observations) before and after the 1974 eruption activity. Because tree rings are formed until the end of August, the NIR-signal was probably not sustained during the growth season of the Monte de Fiore trees. Seismicity started in September 1973, which coincides well with the time of NIR measurement. The figure was created using Adobe Illustrator^64^.

In September 1973, a reduced NIR reflectivity was detected on the western flank of Mt. Etna^27^ at a location where four months later the 1974 eruption occurred. Seismicity, renewed activity at the central vent^37^,^48^ and remote sensing historical datasets suggest that magma was intruded into the western flank of the volcano months before the January 1974 eruption (Fig. 6). The NIR signal may have been caused by the soil moisture due to gas condensation near the surface, which reduced the NIR reflectance. Such a positive change of the ecological settings would have had a positive influence on tree growth if it had been sustained during the growth season. However, the analyses of the ring widths of tree samples collected in the Monte de Fiore area indicate that, if volcanic activity was indeed causing the anomalous NIR signal, the pre-eruptive period of the 1974 event started by the end of August 1973 at the earliest. This would imply that the duration of the pre-eruptive activity was of the order of a couple of months, which is well compatible with lengths of other pre-eruptive periods observed at Mt. Etna: 6 days in July 2011 before the start of the south flank event^5^, only weeks in in autumn 1985^71^, in 1989^44^,^46^ and in 1998^3^, while on some occasions lasting months before the onset of eruptions^3^,^37^,^40^,^44^,^45^,^46^. Our study has shown that tree-growth histories can help dating the occurrence of early magma intrusions, and that analyzing tree rings can provide valuable information on pre-eruptive volcanic processes. We therefore encourage the use of such tree-ring analyses when reconstructing the processes involved in past volcanic events.

## Materials and Methods

### Tree ring width analysis

Trees were sampled at 1.3 m height using a 0.5 cm diameter corer 40 cm in length. To account for variability in individual tree growth, two cores were taken from opposite sides of each tree (180° from each other). Each core was glued on a wooden support and prepared by sanding the surface with decreasing grain-size sandpaper so that the tree-ring width could be dated and measured accurately.

We used a Leica Wild M32^TM^ binocular microscope (25–50x magnification) connected to a LINTAB measuring device, coupled with a computer running TSAPwin (Time Series Analysis Program) software (RinnTech, Heidelberg, Germany), to measure the ring widths of our samples with a resolution of 0.01 mm. To assess the quality of the single measurements of the two cores extracted from each tree and to assure that no rings were missing, we crossdated the ring-width patterns of each tree sample-pair visually (TSAPwin) and statistically (COFECHA)^72^,^73^. On average, all series cover a period longer than 70 years (see chronology statistics in Table 1).

### Seismicity levels

During the autumn of 1973, the west flank of Mt Etna was the place of an intense seismic activity. The analysis of the daily seismicity rates at the Mt. Vetore seismic station suggest that seismicity in September-October 1973 was either larger than the seismic activity of late January 1974 or the events were located closer to the seismic instrument. We favor the second hypothesis as the Catania station was detecting less activity in October than in January. During this short seismic survey (10.11–15.11.1973), it was determined that 1) the strongest signals were detected in the western quadrants (stations GVD, VTD, and DNZ and 2) the weakest ones were recorded between East and North directions (stations MSL, CVD, MAR and BVC). The source of the October-November period correspond well to the location of the western flank. The spatial analysis made there could not be supported by other permanent stations already installed at the time along the flanks of the volcano. At the start of the 1974 eruptive period, the seismic sensors located at Serra La Nave (37°41′30″N, 14°45′22.9″E)^74^ were not functioning since the 5th December 1973^44^.

## Additional Information

**How to cite this article:** Seiler, R. *et al*. Tree-ring width reveals the preparation of the 1974 Mt. Etna eruption. *Sci. Rep.* **7**, 44019; doi: 10.1038/srep44019 (2017).

**Publisher's note:** Springer Nature remains neutral with regard to jurisdictional claims in published maps and institutional affiliations.

## Supplementary Material

Supplementary Dataset 1

## Figures and Tables

**Figure 2:**
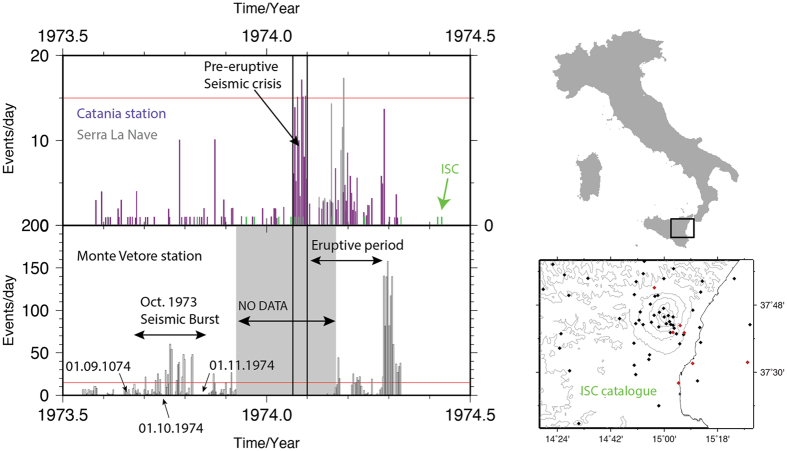
Seismic activity. Seismic activity as detected by seismic sensors deployed on the flanks of Mt. Etna and its surroundings between the summer 1973 and the summer 1974. International Seismological Center (ISC) data is shown with green bars, daily rates of seismicity measured at different stations are grey and purple^37^,^44^. The black dots are events between January 1970 and January 1980 and the red dots are events between 1973.6 and 1974.6 from ISC.

**Table 1:** Sample overview information.

*site*	*no. of trees*	*no. of cores*	*length (yr*)	*ser. interc.*	*mean length (yr*)	*elevation (m a.s.l.*)	*species*
Control	**50**	**76**	**195**	**0.529**	**82.7**	~1600	*Pinus nigra*
Monte de Fiore	**26**	**52**	**130**	**0.597**	**95**	~1700	*Pinus nigra*
*MFn*	*10*	*20*	*99*	*0.660*	*72.3*		
*MFs*	*16*	*32*	*130*	*0.614*	*109.3*		

Description of each dataset (Control and Monte de Fiore south, MFs, and north, MFn) in study, with number of trees and cores, total length of group chronologies, series intercorrelation (ser. interc. = measure of common growth signal in the chronology), mean sample length, elevation and species.
