# Supplementary material for: Tree-ring width reveals the preparation of the 1974 Mt. Etna eruption
Source: Sci Rep. 2017 Mar 7;7:44019. doi: 10.1038/srep44019 (PMC5339818; doi:10.1038/srep44019)
Supplement: Supplementary Dataset 1 [file srep44019-s1.doc]

**Supplementary Materials**

**Manuscript title: Tree growth reveals the development of the 1974 Mt. Etna eruption**

**Authors:** Ruedi Seiler, Nicolas Houlié and Paolo Cherubini

Supplementary Fig. S1 **MF chronologies and sample replication:** Sample replication in MFn (a) and MFs (b). The time when most trees germinated between 1910 and 1920 in MFs and in the 1940s - 1950s in MFn indicates that germination must have happened after an event such as a wildfire or clear cut.

Supplementary Fig. S2 **Tree-growth patterns:** Comparisons of the detrended ring-width chronologies of Monte de Fiore and control trees. Growth patterns differed little before the eruption in 1974 (dotted green line), but the Monte de Fiore chronology shows that the ring-width was greatly reduced after the eruption (1974-1975) probably due to tree growth disturbed by the heat of the superficial lava streams.


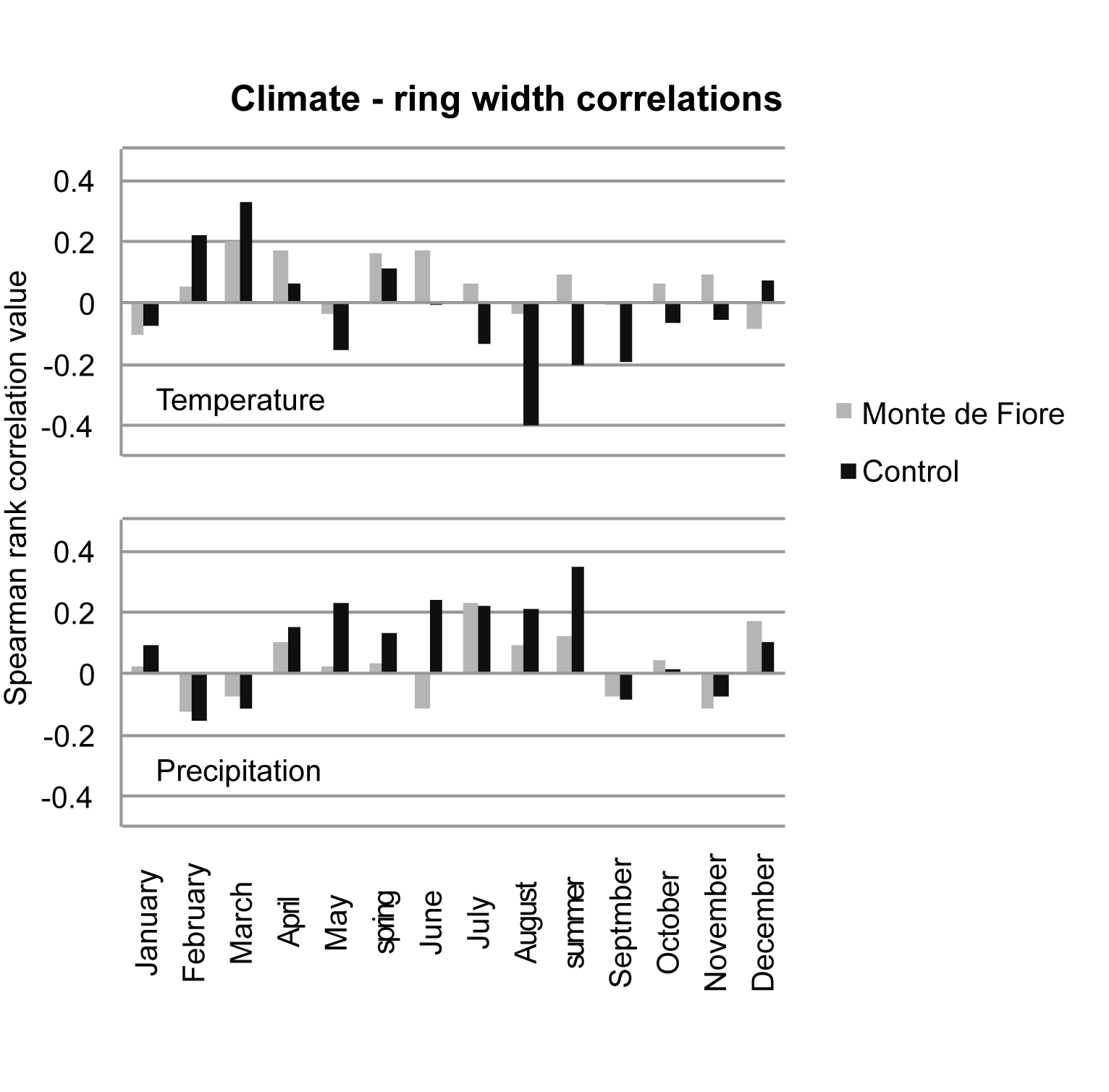


Supplementary Fig. S3 **Climate - ring width correlations:** Spearman rank correlation coefficients for ring-width and climate anomalies (avg = average and tot = total) show low climatic influence on tree-growth at Monte de Fiore (black). The only significant correlation at Monte de Fiore was found between ring width and precipitation in July (r = 0.225; p < 0.05), but climate had a stronger influence on ring width in the control site at Piano Provenzana (grey) with significant correlation values (p < 0.05) for temperatures in March (r = 0.33), August (r = -0.4) and precipitation in May (r = 0.23), June (r = 0.24) and July (r = 0.22).


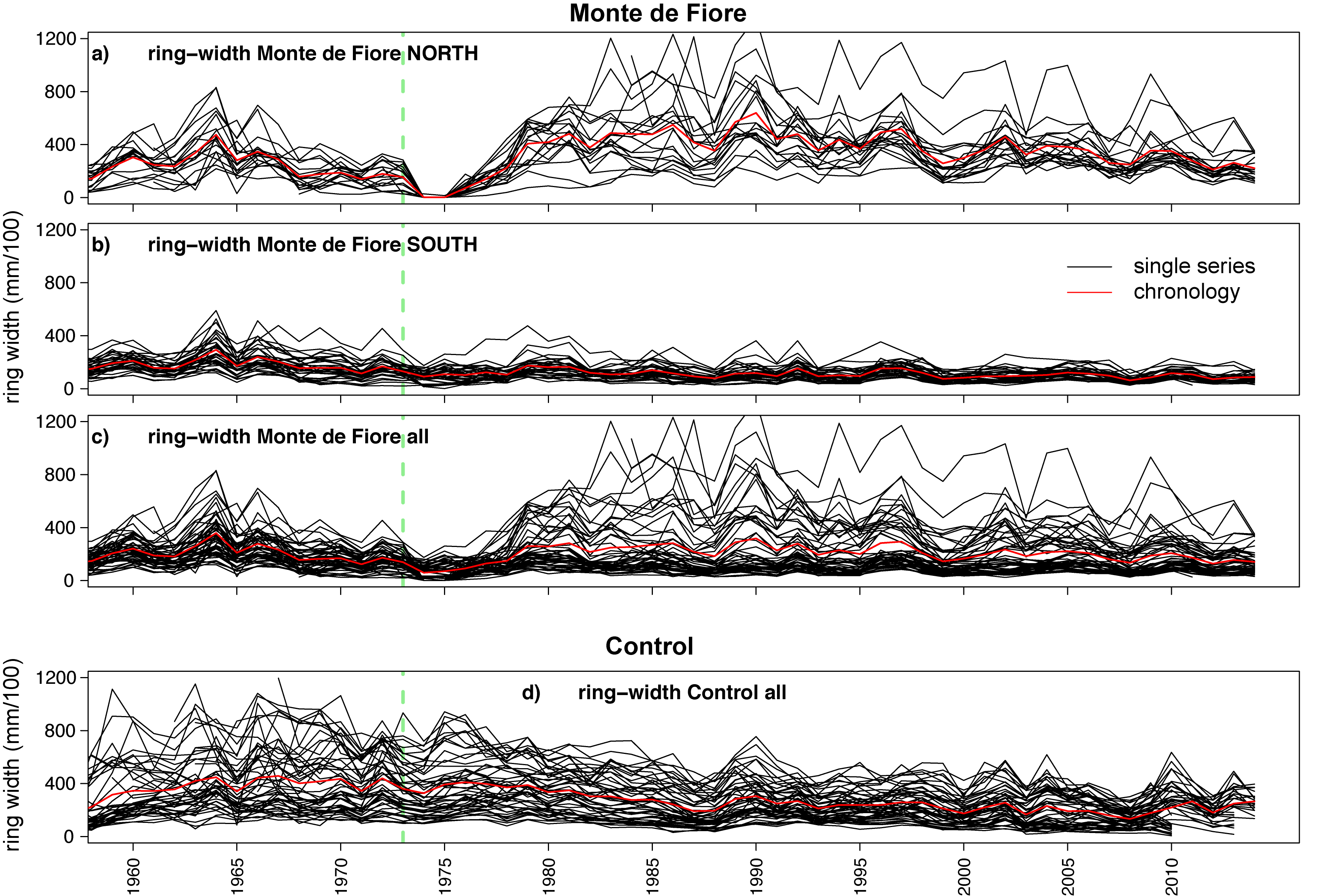


Supplementary Fig. S4 **Raw ring width before and after the 1974 eruption:** Ring-width series (black) and group chronologies (red) for all Monte de Fiore trees (a), the northern sampling patch (b, MFn), the southern sampling patch (c, MFs) and all control trees (d).
